# The Neuroanatomy of the Habenular Complex and Its Role in the Regulation of Affective Behaviors

**DOI:** 10.3390/jfmk9010014

**Published:** 2024-01-03

**Authors:** Jordan Allan Piper, Giuseppe Musumeci, Alessandro Castorina

**Affiliations:** 1 School of Health Sciences, College of Health and Medicine, University of Tasmania (Sydney), Sydney, NSW 2040, Australia; jordan.piper@utas.edu.au; 2 Laboratory of Cellular & Molecular Neuroscience (LCMN), School of Life Sciences, Faculty of Science, University of Technology Sydney, P.O. Box 123, Sydney, NSW 2007, Australia; 3 Department of Biomedical & Biotechnological Sciences, Anatomy, Histology & Movement Sciences, University of Catania, 95123 Catania, Italy; g.musumeci@unict.it

**Keywords:** habenula, dorsal diencephalic conduction system, affective behavior, anti-reward system, depression, anxiety, parental behavior

## Abstract

The habenular complex is a diencephalic structure divided into the medial and lateral divisions that lie within the epithalamus of most vertebrates. This brain structure, whose activities are mainly regulated via inputs/outputs from and to the stria medullaris and the fasciculus retroflexus, plays a significant role in the modulation of anti-reward behaviors in both the rodent and human brain. Such anti-reward circuits are regulated by dopaminergic and serotonergic projections with several other subcortical and cortical regions; therefore, it is plausible that impairment to this key subcortical structure or its connections contributes to the pathogenesis of affective disorders. Current literature reveals the existence of structural changes in the habenula complex in individuals afflicted by such disorders; however, there is a need for more comprehensive investigations to elucidate the underlying neuroanatomical connections that underpin disease development. In this review article, we aim to provide a comprehensive view of the neuroanatomical differences between the rodent and human habenular complex, the main circuitries, and provide an update on the emerging roles of this understudied subcortical structure in the control of affective behaviors, with special emphasis to morbid conditions of the affective sphere.

## 1. Neuroanatomy of the Habenular Complex

**Figure 1:**
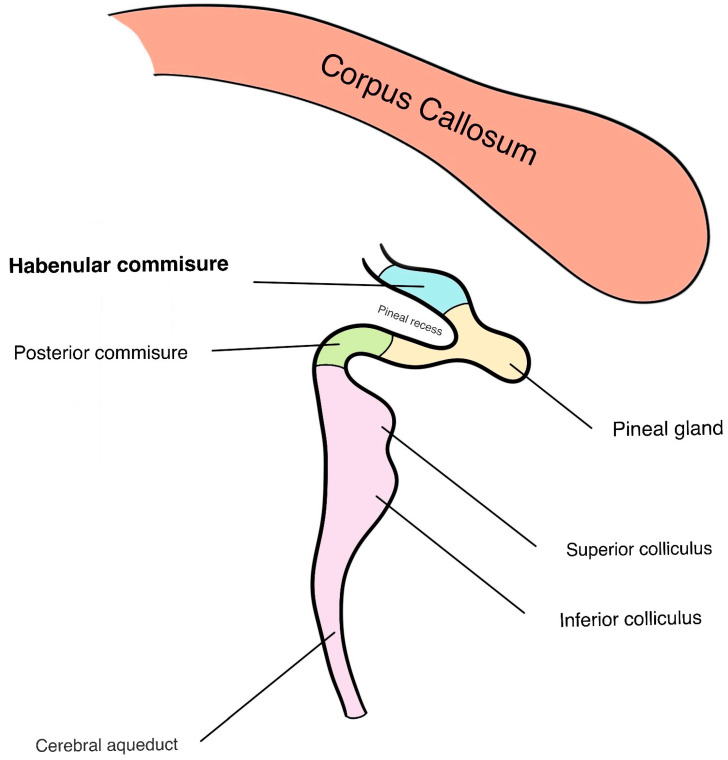
Schematic illustration demonstrating a mid-sagittal view of a typical human brain with a focus on the caudal structures. Note the tectum, including both the superior and inferior colliculi protruding caudally in purple. Dorsal to that, the posterior commissure (in green) extends to the pineal gland (in yellow). The pineal gland continues rostrally as the habenular commissure (in blue), which serves as the articulation point between both the left and right habenula in the habenular trigone.

The habenula is a complex of nuclei, partitioned into a medial and lateral division, both of which are in the dorsal diencephalon. Along with the pineal body and the stria medullaris (SM), the habenula complex is considered to be a component of the epithalamus [1]. The structure of the habenula, much like other diencephalic structures, is characterized by its high degree of conservation and phylogenetic antiquity, making it a prominent feature in most vertebrate species [2,3]. In humans, the habenula, which measures about 5–9 mm in diameter with a total volume of 30–36 mm^3^ [4], sits within a small triangular depression in the wall of the third ventricle known as the habenular trigone. This region, positioned dorsal to the tectum and medial to the midline thalamic nuclei, serves as an extension of the pineal gland [5]. The habenula nuclei are structurally connected between the two trigones by means of the habenular commissure, a white matter tract rich in GABAergic terminals [6]. Located within the superior lamina of the pineal gland stalk, the habenular commissure is visible on the mid-sagittal plane (Figure 1) and serves as a reliable landmark to approximate the habenular nuclei deep into it [7]. Despite their bilateral presence, the habenular nuclei tend to demonstrate directional asymmetries between the right and left sides, a variation not often found in other diencephalic structures [8]. Specifically, the habenular volume demonstrates left-side predominance in both male and female humans, primarily attributed to the enlargement of the left lateral habenula (LHb) [9]. Remarkably, the LHb makes a significant contribution to habenular size, accounting for approximately 95% of the total volume, which distinguishes it from other mammals [9]. In contrast, the volume of the medial habenula (MHb) exhibits no significant left-right asymmetry in either sex among humans. Given the large contribution the LHb makes to the habenular complex volume, this review will focus mainly on the functional and behavioral implications of the lateral division (LHb), with comparative anatomical analysis being made mainly between the rodent and human habenula.

**Figure 2:**
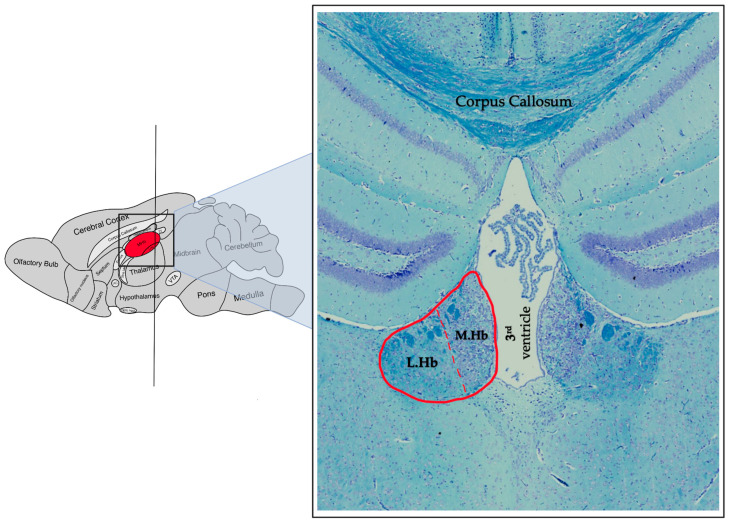
A representative photomicrograph of a coronal mice brain section stained with Luxol fast blue/Cresyl violet showing the habenular complex (delineated in red). On the left side is a schematic indicating the approximate antero-posterior location of the coronal section (right side). Please note the dotted red line highlighting the division line separating the lateral habenula (LHb) and the medial habenula (MHb). Please also note the different neuronal density (neurons are stained in purple) and size of these structures.

**Figure 3:**
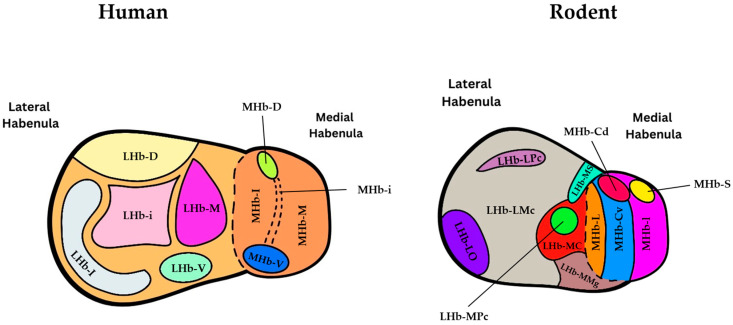
Schematic illustrating the subnuclear organization of the habenular complex in both humans (**left**) and rats (**right**) on coronal sections according to [18,19]. In rats (**right**), the abbreviations for the different subregions of the habenula are as follows: **MHbI**: Inferior part of MHb, **MHbL**: Lateral part of MHb, **MHbS**: Superior part of MHb, **MHbCv**: Ventral region of the central part of MHb, **MHbCd**: Dorsal region of the central part of MHb, **LHbMC**: Central part of the medial division of LHb, **LHbMMg**: Marginal part of the medial division of LHb, **LHbMPc**: Parvocellular part of the medial division of LHb, **LHbMS**: Superior part of the medial division of LHb, **LHbLPc**: Parvocellular part of the lateral division of LHb, **LHbLO**: Oval part of the lateral division of LHb, **LHbLMc**: Magnocellular part of the lateral division of LHb. Note that the rodent basal part (**LHb-LB**), marginal part **(LHb-LMg)** and and anterior part **(LHb-MA)** of the LHb are not shown in this figure. In humans (left), the abbreviations for the different subnuclei of the habenula are as follows: **LHb-d**: Dorsal subnucleus of LHb, **LHb-l**: Lateral subnucleus of LHb, **LHb-i**: Intermediate subnucleus of LH, **LHb-v:** Ventral subnucleus of LHb, **LHb-m**: Medial subnucleus of LHb, **MHb-d**: Dorsal subnucleus of MHb, **MHb-v**: Ventral subnucleus of MHb, **MHb-i**: Intermediate subnucleus of MHb, **MHb-m**: Medial subnucleus of MHb, **MHb-l**: Lateral subnucleus of MHb.

The rodent habenula, similar to that of the human, is divided into both a medial and lateral component, both of which are located dorsal to the thalamus and in close proximity to the 3rd ventricle (Figure 2). In the mouse, the Hb measures approximately 0.8 mm [10] in height and width and is also connected by the habenular commissure on either side [11]. It is worth noting that whilst humans exhibit notable volumetric left-right asymmetry in the habenula complex, this phenomenon is much less pronounced in the mouse brain [12]. Nevertheless, when we compare the subnuclear organization in rodents to that in humans, a distinct difference becomes apparent. Specifically, there are notable differences in the shape, relative size, and intranuclear organization of both the LHb and MHb (Figure 3) [5].

Distinguished by its notable dorsal enlargement, the human LHb sets itself apart from rodents in a striking manner (Figure 3). Not only is the ratio of LHb to MHb volume five times larger when compared to rats, but the relative area occupied by fibers entering the habenula through the SM is also substantially greater [5]. Whilst this disparity may suggest a reduced engagement of striatal and limbic afferents within the LHb of rats [5], it is intriguing to note that the analysis of the rat LHb cytoarchitecture reveals a significant degree of morphological heterogeneity [13]. Unlike humans, however, early light and electron microscopic findings supported the idea that the rat habenula houses 15 subnuclei, 5 in the medial and 10 in the lateral division, which was later substantiated by further electrophysiological and immunohistochemical studies [14,15]. This neuroanatomical configuration suggests that the subnuclear structure of the MHb demonstrates the overall similarity between humans and rats whilst providing evidence for the distinct variations in shape, size, and intranuclear organization of the LHb, thereby highlighting a species-specific structural difference of this brain region [11]. However, emerging transcriptional studies have identified distinct and spatially defined neuronal cell populations in the two habenular divisions that seem to engage differently during aversive stimuli and exhibit differing electrophysiological properties [16,17]. The two ventral MHb subtypes were found to co-express transcripts for glutamate and acetylcholine neurotransmission, with potential implications in aversive memory formation and nicotine addiction.

The study also explored the dorsal and superior MHb subtypes, revealing a high expression of certain genes but highlighting the need for further analysis to understand their function and contribution to neurotransmitter release. In the LHb, the research builds on recent studies to map subnuclei based on differentially expressed genes (DEGs) from single-cell transcriptional profiling. It identifies various DEGs pertaining to G protein-coupled receptors (GPCRs) in LHb neurons, suggesting these as potential therapeutic targets for conditions such as depression, anxiety, and addiction [16].

In another study, using a similar single-cell RNA sequencing (scRNAseq) approach, researchers investigated the cellular diversity of neurons in the two main habenular subdivisions of mammals. The scRNAseq analysis revealed 12 distinct neuronal clusters across the MHb and LHb. Within the MHb clusters, Tachykinin 2 (Tac2) expression marked all six clusters, while classical markers choline acetyltransferase (Chat) and Slc18a3 (which encodes for the vesicular acetylcholine transporter 3) were expressed in only three clusters. Tac1 and Calbindin 1 (Calb1) were expressed in the remaining orthogonal MHb clusters. Reciprocal connections between the LHb and raphe serotonergic system were reflected in the enrichment of Htr2c in half of the six LHb clusters, marked by Protocadherin 10 (Pcdh10), with GABA transporter 1 (Slc6a1) and Calcium Voltage-Gated Channel Auxiliary Subunit Alpha2-Delta 1 (Cacna2d1) marking the other clusters [17].

Interestingly, the study found that while all clusters exhibited enriched DEGs, few clusters had exclusively selective genes. Most marker genes were discriminative and expressed in multiple clusters. To further explore this, the researchers performed HiPlex FISH, expanding the conventional three-color FISH to detect over 10 discriminative genes sequentially. The HiPlex FISH largely corresponded with scRNAseq clusters in both MHb and LHb, demonstrating spatially biased distributions.

Anatomical divisions of the MHb and LHb into subnuclei were considered, and HiPlex FISH analysis suggested that some transcriptionally defined clusters corresponded with anatomically defined subnuclei while others were distributed across or within subnuclei. For example, cells in certain MHb clusters were located in different parts of the MHb subnuclei. Comparison with the study by Wallace et al. [17] showed consistency in some marker genes’ distribution but revealed finer cell types in this study, possibly due to technological differences.

In conclusion, these two recent investigations provided a detailed analysis of the molecular and cellular diversity within the MHb and LHb using advanced sequencing and imaging techniques, contributing to a comprehensive understanding of habenular cellular diversity. Future directions include integrating datasets from different studies to further enhance the understanding of habenular neurobiology. These aspects highlight some important differences in the activities driven by the multiple inputs received by the LHb and MHb divisions, although they still warrant further investigation.

## 2. Main Input and Output Circuitries

**Figure 4:**
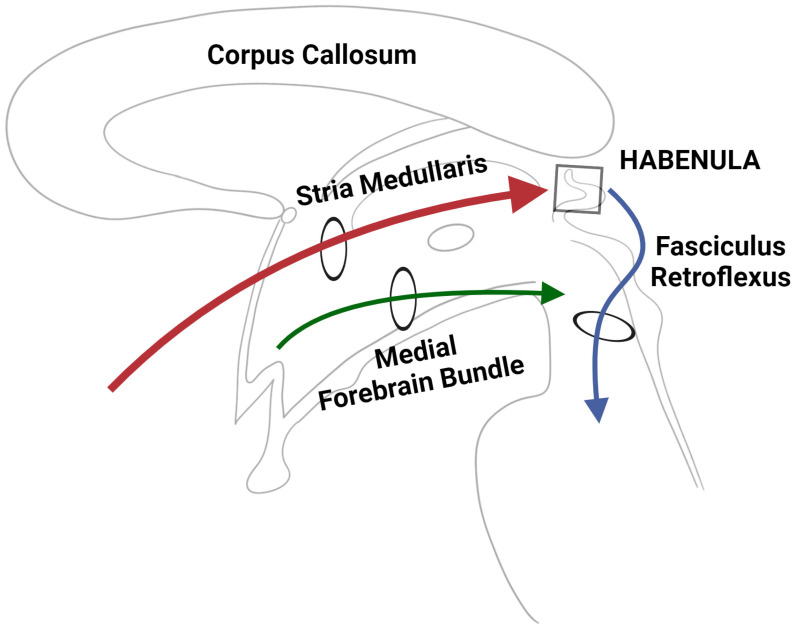
A simplified schematic depicting the three major input/output circuitries of the human habenular complex.

Historically, the habenular complex has been divided into its most simplistic functional components, that is, a limbic component and a motor component. The exact circuitries that govern the functions of these habenular nuclei, however, are far more complicated and involve many intricate and complex intersecting pathways. Three such pathways, all of which are well known for their role in coordinating the major input/output circuitries of the habenular complex, are the SM, the fasciculus retroflexus (FR), and the medial forebrain bundle (MFB) (Figure 4) [20]. These three main pathways allow communication to and from the habenula via a complex but functional network of connections. Interestingly, these connections appear to be partly regulated by the habenula itself. In fact, an elegant study has pinpointed how the habenular subnuclei (especially those within the lateral division) send out efferent projections that determine their own afferent signals, an inherent regulatory mechanism that seems to be needed to control especially dopaminergic inputs to the habenular nuclei [21]. Given the well-established role of the habenula in regulating midbrain monoaminergic systems and integrating cognitive and emotional processing, it seems reasonable that most of these pathways extend to the brainstem and cortex, in addition to basal and limbic systems [22]. The SM and the FR are considered to be two components of what is known as the diencepahlic conduction system, a highly conserved pathway linking the basal forebrain and the monoaminergic brainstem by means of the habenula [23].

**Figure 5:**
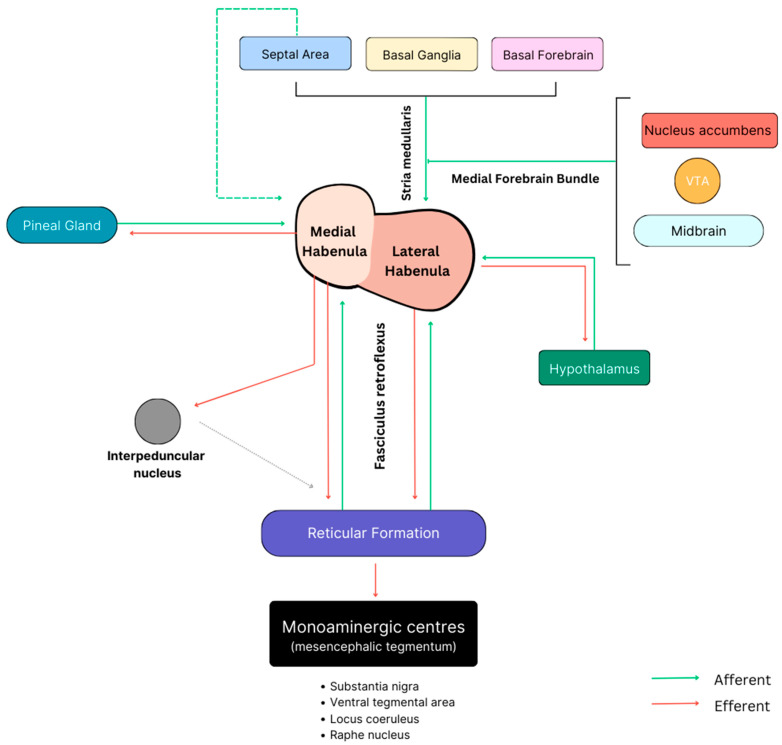
A theoretical schematic representing the major input and output circuitries to both the MHb and the LHb in humans. Note that the three pathways governing most circuitries involve the SM (**right**), the FR (**below**), and the MFB (**above**).

As depicted in Figure 4, the stria medullaris (SM) is a significant bilateral bundle of axonal fibers found in the epithalamus, serving as the principal pathway for input to the habenula. This bundle primarily consists of afferent fibers originating from various regions of the hypothalamus, basal nuclei, basal forebrain, and the septal area [24]. Interestingly, the stria medullaris constitutes a smaller percentage of the cross-sectional area of the habenula in rats (12%) compared to humans (30%), highlighting a notable disparity in the SM-habenular interface between the two species [5]. Given its considerable size, it is easily observable on a gross mid-sagittal section, forming a horizontal ridge on the medial surface of the thalamus. It extends from the anterior commissure, passes dorsal to the interthalamic mass in the roof of the 3rd ventricle, and continues caudally before terminating at the habenula [25]. Unlike the lateral fibers of the SM, the medial fibers decussate at the habenular commissure before passing to the respective contralateral habenula [5,26]. Contrary to its initial association with the olfactory system [1], recent research has revealed that the stria medullaris functions as the primary input circuit within the behavior-modifying diencephalic conduction system [23]. The way in which it does this is by collecting afferent fibers from the septal area, basal forebrain, basal ganglia, and hypothalamus [22,27] before transmitting this information in a (predominantly) unilateral manner before targeting the LHb (Figure 5). The largely cholinergic, glutamatergic, and GABAergic nature of these afferents underscores their potential involvement in a diverse array of neuropsychiatric disorders, particularly in the event of deficits within the SM. For the same reason, restoring SM functionality using deep brain stimulation may also serve as a therapeutic target for the treatment of certain affective behaviors [28].

The fasciculus retroflexus, similar to the SM, is a bidirectional white matter tract, forming the final component of the dorsal diencephalic conduction system [23]. However, the fibers from this tract, unlike the SM, are predominantly efferent and serve as the bridge between the habenula complex and the monoaminergic centers in the brainstem [29] (Figure 4 and Figure 5). Although the specific trajectory of this tract is not fully understood, it is widely recognized that some of the contributing fibers originate from the ventral region of the LHb before descending through the caudal thalamus [10]. As it continues ventrally in front of the pretectal area, it reaches the basal plate and undergoes a notable 90-degree caudal turn to enter the interpeduncular nucleus (IPN) via its rostral and dorsal borders [23] (Figure 5). This inversion of trajectory, characterized by a sharp bend, is what confers its unique name, “retroflexus”, which literally means “curved backwards”. The interpeduncular nucleus contains descending projections to the monoaminergic regions, as recently reported by Ables et al. [30], and is recognized for its extensive distribution through the FR, as highlighted by [23]. Whilst the specific downstream nuclei influenced by the FR remain uncertain, there is a consensus that it exerts inhibitory control over monoaminergic centers via its GABAergic fibers [31,32].

Lastly, the medial forebrain bundle, a small but remarkable white matter tract containing mostly unmyelinated nerve fibers, acts as the principal route between the limbic forebrain and the midbrain (Figure 4). Given its origin in the olfactory regions, it is plausible that this pathway is significantly enlarged in those mammals with more pronounced olfactory guidance systems, such as in rodents; however, it is important to note that this tract still remains dominant in non-human primates to this day [33]. Whilst the MFB does not project directly to the habenula, it does, however, make secondary projections to the LHb via the convergence of collateral fibers onto the SM [1]. The existence of such connectivity overlaps between the SM and the MFB and implies the potential for functional parallelism between the two neural pathways [1,34]. The complexity of the MFB is extensively documented in the literature, which identifies approximately 50 fiber sub-components and up to 13 distinct neurotransmitters associated with it [26,35,36]. Due to its inherent complexity, traditional imaging studies have proven to be inadequate in unraveling its exact trajectory and connections. It was only with the adoption of tractography that some of the circuitry within this tract became truly apparent. One such study revealed that extensive connectivity exists between the ventral tegmental area (VTA) and reward-related subcortical and cortical prefrontal regions [37]. It has also been documented that the projections from the MFB into the habenula mainly arise from the VTA, the prefrontal cortex, and some components of the limbic system (Figure 5). Due to its extensive connectivity and involvement in multiple neural systems, the MFB is considered a significant convergence point for regulating motivation and coordinating effector systems [1].

Unlike the afferent fibers, the efferent fibers of the lateral habenula are extensively distributed and serve many brain regions primarily through both the FR and, to a certain degree, the MFB [1,23]. As the LHb tends to receive the majority of its input from the forebrain, limbic, and basal ganglia circuits, its outputs are directed mainly to the brainstem, where it helps govern behavioral inhibition [38]. Specifically, the efferents from the LHb primarily direct their influence on midbrain nuclei, encompassing the dopaminergic VTA and substantia nigra, in addition to GABAergic rostromedial tegmental nucleus and the serotoninergic dorsal and median raphe nuclei [3]. It is by means of this system that frontolimbic regions can regulate the release of brainstem monoamines through the FR, thus controlling the monoaminergic tone [39].

## 3. The Anti-Reward Properties of the Habenular Complex

While the conventional reward system is responsible for activating circuits associated with positive reinforcement and hedonic valence, an anti-reward system also exists that suppresses reward by promoting actions that discourage behaviors that have led to its activation. Simply, the notion of ‘anti-reward’ stems from the hypothesis that the brain has mechanisms in place to limit the effects of reward [40]. This system is particularly useful in avoiding or preventing actions that can lead to pain, negative emotional associations, and/or perceived threats. In general, the system acts by counterbalancing the properties of the typical reward circuits. In a canonical reward circuit, dopaminergic neurons in the VTA are stimulated to induce a surge of dopamine via one of the reward pathways: the mesocortical, mesolimbic, nigrostriatal, or the tuberoinfundibular [41]. Of those pathways, the mesolimbic pathway tends to be most often recruited in typical reward circuitries [42]. As dopaminergic stimuli are transported via this pathway, they promote the release of dopamine, which is modulated by the nucleus accumbens in the ventral striatum and prefrontal cortex to encourage positive reinforcement of behaviors associated with the anticipated reward [43]. In contrast, the anti-reward system involves primarily the LHb and two pathways that connect it with the VTA: the direct pathway and the indirect pathway. The direct pathway, as the name implies, spans from the LHb to the VTA, whilst the indirect pathway projects from the LHb to the VTA via the rostro-medial tegmental nucleus [44]. Tracing studies, in addition to immunocytochemistry and in situ hybridization experiments, have revealed that whilst most LHb neurons are glutamatergic, their axons terminate onto GABAergic neurons in the ventral midbrain via these pathways [45]. In other studies, electrical stimulation of the LHb in non-human primates and rats demonstrated noticeable inhibitory effects of dopaminergic neurons in the midbrain and caused anhedonia [46,47]. These findings suggest that the LHb acts in opposition to conventional reward circuits by promoting inhibitory control over dopaminergic neurons and is, therefore, involved in anti-reward processing [48,49]. An early study in 2007 reaffirmed this notion when neurons in the LHb became activated after monkeys failed to receive an expected award or when the animal received a negative outcome [46]. Similar outcomes have also been reported in rats [49]. In humans, it has also been observed that the habenula, particularly the left division (and to some degree the right division), exhibited significant activation in anticipation of an aversive event, such as the expectation of receiving small electrical shocks when compared to neutral outcomes [50]. The same study also demonstrated an augmentation in functional connectivity between the VTA and the LHb in response to aversive stimuli, providing additional support for the existence of anti-reward circuitry connecting these regions. Consistent with evidence from rodent models, these findings support the idea that the LHb exerts control over signals with a negative motivational salience that is transmitted to the midbrain dopaminergic system [22,46,51]. More recent work has also demonstrated that the human LHb exhibits sensitivity to probabilities and tracking prediction errors. Notably, when certain cues anticipate a greater probability of point loss in a guessing game, significant activation is observed in the left-sided LHb [52]. Conversely, neuroimaging studies have demonstrated that heightened adversity of anticipatory cues primarily triggers activation in the dominant right-sided LHb, highlighting lateralization in the processing of the motivational salience of these cues [50,53]. Not surprisingly, the human habenula tends to exhibit increased activation for punishment-related stimuli when compared to reward-related stimuli, a similar finding to what is reported in non-human primates [46]. Interestingly, a high-resolution brain mapping study revealed asymmetries in the functional connectivity of the habenula in humans [54]. However, habenular lateralization in connectivity patterns does not correlate with anatomical asymmetry, given the vast structural heterogeneity seen in brain mapping studies. In fact, although at least one study suggests that smaller right-side habenula volume is associated with major depressive disorder, imaging data from a meta-analysis conducted on 1427 patients with either depression, schizophrenia, and healthy controls demonstrated that while inversed asymmetry patterns were suggested in patients with depression (leftward) and schizophrenia (rightward), no significant disorder-related differences relative to healthy controls were found in either the left-right asymmetry or the unilateral volume of the habenula [25].

## 4. The Role of the Habenular Complex in Affective Disorders

Affective disorders are those pathological conditions that are characterized by a change in mood or affect [11]. Due to “mood” being regulated via a series of well-orchestrated changes to brain connections, neurochemistry, and electrical activities within the central nervous system (CNS), the pathogenesis of mood-related disorders is both complex and multifaceted. What is known, however, is that both the amygdala and the orbitofrontal cortex play a significant role in the modulation of emotional processes [55]. In view of the contribution of the habenular complex in modulating the interplay between these limbic structures and the midbrain to control the monoaminergic influence on telencephalic structures, it is reasonable to suggest that the habenula is vital in the development of specific affective disorders, including depression, stress-induced anxiety, and nociception [11]. More specifically, given the input the LHb receives from limbic structures such as the cingulate gyrus and the insular cortex in the rat [38], it is probable that the LHb plays a substantial role in the genesis of anxiety-related disorders and the perception of pain. Moreover, recent studies have highlighted the potential involvement of a hyperactive subgenual cingulate gyrus in the pathogenesis of depression [56,57]. In this context, the activation of the Descending Dopaminergic Control System—through the collaboration of the LHb, VTA, and nucleus accumbens (NaC)—is suspected to play a role in attenuating these hyperactive states by modulating relevant extrapyramidal re-entry circuits, contributing to mood stabilization.

### 4.1. Depression

Limbic system abnormalities have long been implicated in the pathogenesis of many affective disorders; however, the habenular complex and its role in most of these disorders remains relatively ambiguous [11,58,59]. As previously highlighted, the LHb promotes anti-reward properties, an evolutionary-conserved mechanism that contributes to species survival by inhibiting those behaviors that result or are predicted to result in a negative outcome [60]. The way in which the habenula contributes to this function is thought to involve the inhibition of dopaminergic activity within the VTA [46]. In normal conditions, the LHb is activated by a range of negatively associated emotional stimuli and stressors; however, excessive stimulation of the LHb is also believed to contribute to the development of depressive states, possibly due to its excessive inhibition of monoaminergic centers [61]. Early studies reiterated this point by demonstrating that the depletion of tryptophan, the precursor to serotonin, reduced the connectivity of the habenula-raphe pathways [62]. This finding not only suggested that disruption to the habenular complex can interfere with the serotoninergic activity in cortical and subcortical regions but also shed light on the possible link between the habenular activity and the severity of depression [62]. This early finding was later supported by Ranft et al., who demonstrated that there was a significant reduction in the LHb volume in those patients who suffered from depression when compared with healthy controls. In the same study, the authors also reported quantitative changes in neuronal cell populations within the right-sided habenula of depressed patients [11]. Rodent models of depression added to this understanding by demonstrating enhanced synaptic activity in VTA-projecting habenular neurons in addition to higher metabolic demand [44,63]. Perhaps most interesting is that typical antidepressant treatment showed a remarkable reversal of habenular activity and depressive behaviors in these rodents [61,64].

A very recent study identified the lateral hypothalamus (LH) as the most physiologically relevant input to the LHb under stress [65]. The authors describe the crucial role of the LH-LHb synaptic potentiation in stress-induced depression. These findings are in partial agreement with an even more recent work, where it was found that potentiating GABAergic adenosine A_2A_ receptors (A_2A_R)-positive neurons in the lateral septum or its connections to the dorsomedial hypothalamus (and not the LH) and the LHb is sufficient to phenocopy a depressive-like status in chronically stressed rodents [66]. A further study corroborated the importance of the LH in activating the LHb so that the latter can inhibit the medial prefrontal cortex (mPFC) that controls social competitiveness and reinforces retreats in contests—two core neural mechanisms mutually promoting social status loss and depressive behaviors [67].

By revealing the role of habenular overactivation as an underlying theme in depressive-related disorders, these studies contribute to our understanding of the condition and may shed light on how the mitigation of such overactivation through deep brain stimulation may have the potential to enhance monoaminergic projections to subcortical regions. This offers valuable insights into the mechanism behind the success of this intervention, especially in individuals with treatment-resistant depression [68]. Overall, given that the prevailing hypothesis of disrupted monoamine neurotransmitters acts as a key contributor to the pathophysiology of depression, the current literature strongly suggests that the overactivation of the LHb, concurrent with the inhibition of monoaminergic systems, may, therefore, play a significant role in the pathogenesis of depressive disorders.

### 4.2. Stress and Anxiety

When humans or other sentient species perceive or experience actual threats, the CNS triggers a series of physical, emotional, and intellectual responses to adapt to the change. Whilst this process is physiological, as it prepares the body to accommodate such conditions in the acute phase, it may become debilitating and cause physiological impairment if the condition persists over time. Since the habenula is a key regulator of the anti-reward system, it is not surprising that this structure is activated during stress, especially neurons within its lateral division [69,70]. In one paper, researchers demonstrated that stimulation of the rat LHb induced a vasoconstrictive thermoregulatory response, which is typically seen only during times of emotional stress. This finding reinforced the theory that the LHb plays a significant role in stress signaling [71]. Specifically, the medial subnuclei of the LHb [72], a region receiving strong limbic dopaminergic inputs, seem to be most implicated [34,45,73,74]. As stress-induced anxiety plays a role in modulating motor activity [75], rats exposed to stress exhibit notable activation of LHb neurons, leading to pronounced inhibition of dopaminergic neurons and, thus, suppression of locomotor activity [44]. This reaffirms the notion that stress, via the LHb, inhibits dopamine secretion [75], a finding consistent with previous literature demonstrating that chronic stress causes monoamine and behavioral dysfunctions [76,77]. This concept was later emphasized by Sachs and coworkers [78], who demonstrated that the LHb-driven dysfunctions of monoamines’ secretion can be a substrate for increased susceptibility to stress. On the other hand, there is only scant evidence of the involvement of the MHb in stress responses. In a study where immunotoxin-mediated lesions were inflicted on the MHb afferents arriving from the anterior commissure and triangular septum, disturbances to anxiety and fear-associated behaviors were reported [79]. In other studies, the MHb showed a significant increase in pro-inflammatory cytokines release and mast cell activation following both acute and chronic stress induction in rats [80,81], thus indicating that alterations to MHb pathways and immune activation in this habenular division could be linked with the development of anxiety states under stress conditions. Overall, stress seems to alter the functionality of the LHb, causing inhibition of dopaminergic and serotoninergic outputs, whilst further studies are warranted to better clarify the involvement of the MHb in anxiety/fear responses associated with stress [82].

### 4.3. Nociception

Nociception is “an unpleasant sensory and emotional experience associated with, or resembling that associated with, actual or potential tissue damage”, as defined by the International Association for the Study of Pain (IASP) [83]. The underlying noxious sensory response, which initiates the cascade of nociceptive activation, helps mammals avoid or withdraw from potential or actual tissue damage and is governed via a 3-order neuron chain commencing from the periphery, relayed in the spinal cord to then reach higher cortical areas. What is important to note, however, is that pain is not merely a physical sensation but rather the integrated processing of emotional/physical responses evoked by the initial unpleasant/noxious sensation. As such, nociception is a much more complex experience than physical pain and involves the crosstalk between cortical and subcortical areas that regulate emotions, unpleasant physical sensations, as well as memories of prior painful experiences. The limbic system, the diencephalon, and the cortex are the regions that are mainly implicated in such nociceptive processing [84]. Considering the influence of dopamine and serotonin on pain perception, it is not surprising that the LHb, given its connections with various dopaminergic and serotonergic nuclei, is also a main player in the processing of nociception and the associated emotional experiences. Stimulation of the LHb inhibits dopaminergic activation, and preclinical findings show that LHb neurons only respond to noxious stimuli as opposed to non-noxious stimuli [85]. The habenula has also demonstrated pain modulation properties following intra-habenular and intra-accumbens injections of naloxone. In addition, it was found that naloxone inhibited the analgesic effects of morphine when introduced into the periaqueductal gray (PAG) [86], a midbrain structure functionally connected to the habenula. Similarly, analgesic effects induced by morphine administered into the habenula were inhibited following naloxone administration into the NaC [86]. These findings suggest that the habenula could function as an intermediate relay station, facilitating the transmission of pain modulation signals from the nucleus accumbens to the PAG in descending neurons [87]. The presence of afferent inputs from the lateral hypothalamus, a structure involved in pain modulation [88,89], into the LHb further reaffirms the involvement of the habenula in reinforcing the activation of pain processing pathways [87]. As such, it can be inferred that the habenula may play a pivotal role in the integration of physical, stress-associated, and noxious sensory information, acting in concert with other hubs (i.e., the PAG) to encode, process, and likely reinforce the activation of nociceptive pathways.

## 5. Newly Identified Circuitries Involved in Female Parental Behavior

**Figure 6:**
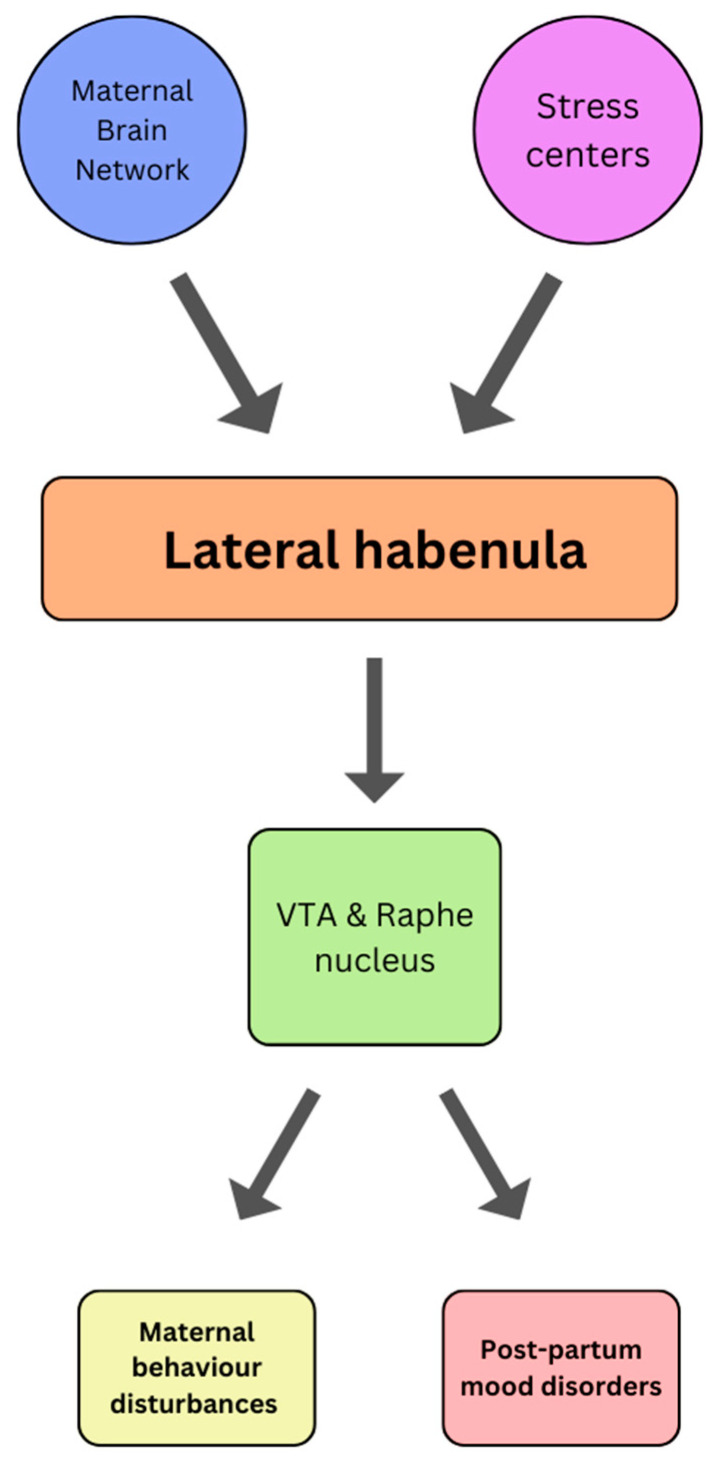
A theoretical schematic representing a novel neurocircuit involving the lateral habenula and its involvement in maternal behavior disturbances and post-partum mood disorders as described by [99]. Note that LHb activity is influenced by the maternal neural systems (such as DRN, VTA, MPOA, and LPOA) and the stress systems (such as PVN, LHA, BNST, mPFC). This is thought to increase excitatory output from the LHb to inhibitory monoaminergic systems in the VTA and DRN. VTA: Ventral tegmental area; LHA: Lateral hypothalamic area; LPOA: Lateral preoptic area; MPOA: Medial preoptic area; DRN: Dorsal raphe nucleus; PVN: Paraventricular nucleus; BNST; bed nucleus of the stria terminalis; mPFC: medial prefrontal cortex.

Parenting is a complex set of behaviors that encompasses a wide range of emotional responses and interactions. Affective behaviors are integral to the parent-child relationship and are crucial for the well-being and healthy development of children. In fact, parenting involves not only meeting the physical needs of children but also addressing their emotional and psychological needs through affective behaviors that promote nurturing guidance and emotional support. As such, the following section will provide a brief overview of the most recent findings pertaining to the role of the habenula in parental behavior. In this respect, there is a growing body of evidence suggesting that the habenula may be implicated in the genesis of certain maternal behaviors. Typically, the act of pregnancy and parturition induce many physiological, chemical, and anatomical changes to the mother’s body, all of which help accommodate the physical, mental, and social demands of parenthood [90]. Of these changes, those affecting the corticolimbic and sensorimotor circuitries are particularly relevant [91]. Structures implicated in these circuitries are the hypothalamus, midbrain, the bed nucleus of the stria terminalis, the prefrontal and parietal cortex, amygdala, PAG, striatum, and cingulate cortex [92]. Together, this interconnected system of structures constitutes the Maternal Brain Network, which is responsible for regulating maternal care and defense [93]. Given the LHb inputs/outputs connecting the median and lateral preoptic area, raphe, PAG, and the VTA [94,95,96,97,98], it is postulated that the habenula plays a role in regulating physiological maternal behaviors whilst also taking part in the development of postpartum behavioral disorders [99]. Interestingly, a discreet percentage of mothers experience postpartum anxiety (5–12%), depression (5–25%), and psychosis (0.1%) [100,101,102], and whilst the pathogenesis of these conditions remains somewhat elusive, there seems to be a strong correlation with postpartum stress [90]. In a very recent preclinical study, virgin female mice exposed to pup distress vocalizations exhibited increased LHb activity and developed aversive behaviors, thus corroborating that a novel neural circuit responsible for modulating maternal behavior exists. Due to the effects of stress on the LHb, it has been hypothesized that maternal stress may disrupt the downstream projections to the VTA and dorsal raphe nucleus and cause suppression of maternal behavior (Figure 6) [99]. Such dysfunction could serve as a mediator between maternal stress and maternal behavior, thus contributing to the underlying pathophysiology of postpartum mental disorders [99]. This theory is particularly credible given that hyperactivity of the LHb is often implicated in both stress and other affective-related disorders, as previously discussed.

## 6. Conclusions

A common theme in the role the habenula plays in the psychopathology of affective behaviors seems to lie with the dysregulation of monoaminergic systems. Given the habenula’s significant role in regulating subcortical regions via the fasciculus retroflexus as well as cortical regions via the stria medullaris, lesions to the habenular complex may, therefore, impose on the dopaminergic and serotonergic projections that are typically involved in the regulation of mood-related processes. Specifically, the literature currently suggests that hyperactivation of the LHb results in significant excitatory output to downstream inhibitory monoaminergic neurons in the VTA and raphe nuclei, thereby resulting in a global reduction in reward-related processing. The absence of this signaling is thought to contribute to the underlying pathogenesis of affective disorders, given that reward-related behavior is reliant on optimal monoaminergic release. For the same reason, however, the LHb has also been implicated in a novel neural circuit that governs maternal behavior due to its involvement in the maternal brain network, a system that also typically requires adequate dopaminergic and serotonergic projections. Given the role the LHb plays in these conditions, it may, therefore, pose as a potential therapeutic target for the treatment of affective disorders via deep brain stimulation. Further investigations that explore the cellular properties of the habenular complex, including the role of the MHb, may help reveal its specific involvement in these conditions.

## Data Availability

All data are reported in the published version of this article.
